# Effect of perioperative use of parecoxib on chronic post-surgical pain in elderly patients after hepatectomy: a prospective randomized controlled study

**DOI:** 10.1186/s40360-021-00501-1

**Published:** 2021-06-15

**Authors:** Xiaodong Ge, Yan Pan, Danfeng Jin, Ying Wang, Shengjin Ge

**Affiliations:** Department of Anesthesia, Zhongshan Hospital, Fudan University, No. 180 Fenglin Road, Shanghai, 200032 China

**Keywords:** Parecoxib, Chronic post-surgical pain, Elderly, Hepatectomy, Multimodal analgesia

## Abstract

**Background:**

Chronic post-surgical pain (CPSP) has a negative impact on the recovery, quality of life, and physical functioning of elderly patients. This study aimed to test the superiority of parecoxib vs. placebo in preventing chronic post-hepatectomy pain in elderly patients under combined general-epidural anesthesia.

**Methods:**

A total of 105 elderly patients undergoing hepatectomy under combined general-epidural anesthesia were randomized into the parecoxib or placebo group. The primary outcome was the proportion of patients with CPSP 3 months postoperatively. The secondary outcomes included the Short-Form McGill Pain Questionnaire score in CPSP-positive responders, acute pain intensity, postoperative analgesic demand, inflammatory markers change, and postoperative complications within 28 days.

**Results:**

The parecoxib group provided a non-significant absolute 9.1% reduction in the rate of CPSP compared to the placebo group (*P* = 0.34). The average chronic pain visual analog scale in the parecoxib group was lower than that in the placebo group (*P* = 0.04). Significantly less moderate-to-severe acute pain at rest (*P* = 0.04) and with coughing (*P* < 0.001), less patient-controlled epidural analgesia (PCEA) consumption (*P* = 0.01), and less rescue analgesia (*P* < 0.001) were observed in the parecoxib group compared to the placebo group. Furthermore, no between-group difference was observed in inflammatory markers (*P* > 0.05) and postoperative complications (*P* = 0.65).

**Conclusions:**

Parecoxib reduced the prevalence of CPSP in elderly patients after hepatectomy under combined general-epidural anesthesia from 44.4 to 35.3% with no statistical significance. Moreover, significantly alleviated CPSP intensity and improved acute pain management were observed.

**Trial registration:**

This study was retrospectively registered in the Chinese Clinical Trial Registry (URL: http://www.chictr.org.cn/edit.aspx?pid=56961&htm=4) on August 3, 2020 (ChiCTR-2,000,035,198).

**Supplementary Information:**

The online version contains supplementary material available at 10.1186/s40360-021-00501-1.

## Background

In China, liver cancer is one of the most commonly diagnosed malignant tumors, mainly treated by surgical procedures. Although liver cancer incidence and mortality trends have been decreasing significantly in recent years [1], China accounts for about half of the world’s new cases each year [2]. Chronic post-surgical pain (CPSP) is a common long-existing postoperative complication and has been studied extensively in various surgeries [3] but rarely in open hepatectomy. For patients after liver transplantation, it was up to 70.5% at 3 months [4]. Approximately half of the elderly patients complain of chronic pain after open hepatectomy during the postoperative follow-up in Zhongshan Hospital, Fudan University. Furthermore, CPSP has no beneficial biological significance in elderly patients and might negatively impact the recovery, quality of life, and physical function [5]. Preventing transformation from acute pain into chronic pain is an essential part of Enhanced Recovery After Surgery (ERAS) [6, 7].

The right subcostal incision is the most applicable method to open hepatectomy. After transection of abdominal muscles and nerves, the incision effectuates the release of inflammatory cytokines and massive initiate cellular reactions to severe tissue injury at the surgical sites [8, 9]. Persistent inflammatory changes enhance peripheral nociceptor sensitivity and sensitize the peripheral and central nervous systems [9]. An increasing body of evidence has shown that neuroinflammation in the peripheral and central nervous system plays a key role in the development and maintenance of chronic pain [8, 10, 11]. Parecoxib sodium is the first selective COX-2 inhibitor for injection, which can partially penetrate the blood-brain barrier and act on the peripheral and central COX-2 simultaneously [12]. Therefore, it exerts an anti-neuroinflammatory effect by inhibiting the synthesis of prostaglandins in both the peripheral and central nervous systems. In addition to the effects on COX-2 pathway, selective COX-2 inhibitors also inhibit the metabolism of endocannabinoids, providing an extra antinociceptive stimulus [9, 13]. Reportedly, parecoxib is beneficial to acute post-surgical pain management [14], but its function on CPSP has not yet been proved. Therefore, we hypothesized that parecoxib prevents CPSP by inhibiting the perioperative inflammatory reaction that facilitated peripheral and central sensitization in elderly patients.

To test this hypothesis, we designed and conducted this prospective randomized controlled study enrolling elder patients over 65 undergoing hepatectomy at the Zhongshan Hospital affiliated with Fudan University. Next, we established a multimodal analgesia system by adding perioperative parecoxib to the routine practice of combined general-epidural anesthesia. Stringent criteria were set to select patients and avoid the potential adverse reactions due to parecoxib. Other potential risk factors for CPSP after hepatectomy were also evaluated in our cohort.

## Methods

### Study design

This prospective, double-blind, random, placebo-controlled, single-centered trial of perioperative analgesia to prevent CPSP in elderly patients after hepatectomy. The objective of the study was to test the superiority of parecoxib vs. placebo in preventing chronic post-hepatectomy pain in elderly patients over 65 under combined general-epidural anesthesia.

### Study participants

All patients, aged 65–80-years-old, with American Society of Anesthesiologists (ASA) physical status classification of I or II, scheduled to undergo elective open hepatectomy for hepatocellular carcinoma at the Zhongshan Hospital, Fudan University, were considered eligible for this study. We excluded patients who underwent hepatectomy previously, had a history of chronic pain, were treated with radiation or chemotherapy, were not suitable for epidural anesthesia (especially with coagulopathy), and had a history of psychology or mental illness. Given the potential adverse reactions of COX-2 inhibitors, patients were excluded if they fulfilled one of the following conditions: 1. allergy to parecoxib; 2. active gastrointestinal bleeding or ulceration; 3. history of congestive heart failure or ischemic cardiac diseases; 4. Child-Pugh score > 6 points or resection of more than three hepatic segments; 5. disease of peripheral arteries or cerebral vessels; 6. estimated glomerular filtration rate of < 60 mL/min.

All participating patients provided written informed consent for this clinical trial, approved by the Ethics Committee of Zhongshan Hospital, Fudan University. A trial registration number (ChiCTR2000035198) was obtained from the Chinese Clinical Trial Registry.

### Randomization and blinding

A computer-generated randomization sequence was used to recruit and enroll patients consecutively. Only the statistician and the pharmacists were aware of the concealed allocation schedule. The participants were randomly assigned to one of the following treatment groups in a 1:1 ratio.

#### Parecoxib group

Parecoxib sodium of 40 mg diluted with normal saline to 4 mL was administered intravenously, starting from 10 min before incision and once every 12 h until the sixth dose.

#### Placebo group

A volume of 4 mL normal saline was administered intravenously, starting from 10 min before incision and once every 12 h until the sixth dose.

Based on the allocation schedule, the unblinded pharmacist prepared the parecoxib sodium or placebo with an identical appearance in the same type of syringe. The nurse (blinded to allocation) followed the study protocol and administered the medication according to the sequence. To reduce the bias, the patients, anesthesiologists, surgeons, nursing staff, postoperative follow-up group, and data processors were blinded to patient grouping until all the data were collected.

### Study procedures

#### Baseline psychological distress

All patients completed a questionnaire of the Hospital Anxiety and Depression Scale (HADS) [15] on the night before surgery.

#### Surgery and anesthesia implementation

All surgical operations were performed through a right subcostal incision by senior physicians with surgical experience of > 5 years. Our institute developed a routine practice of combined general-epidural anesthesia, followed by patient-controlled epidural analgesia (PCEA) in liver surgery. After the patient entered the operating room, a standardized protocol was followed to achieve general anesthesia combined with an epidural block: 1) A central venous catheter was placed through the internal jugular vein to guide intraoperative fluid therapy. 2) An epidural catheter was placed properly at the T8-T9 interval, and the anesthesia plane was tested by 2% lidocaine in a volume of 3 mL. 3) General anesthesia was induced with fentanyl 3 μg/kg, propofol plasma target controlled infusion, and rocuronium 0.6 mg/kg. 4) Intraoperative monitoring was carried out via an electrocardiogram (lead II and lead V5); also, oxygen saturation, arterial blood pressure, central venous pressure, and end-tidal CO_2_ partial pressure were recorded. 5) Anesthesia was maintained by 0.7 minimum alveolar concentration sevoflurane and continuous epidural anesthesia. Intraoperative fentanyl and muscle relaxants were administered on demand.

#### Multimodal analgesia

PCEA pump was applied to each patient after emergence from anesthesia, with the formulation of 0.12% ropivacaine and 2 μg/mL fentanyl. The infusion rate was 2 mL/h, bolus volume was 4 mL, and lock time was 10 min. The patients received parecoxib sodium or placebo intravenously, starting from 10 min before the incision and once every 12 h till the sixth dose. In addition, non-NSAIDs rescue analgesia, according to the surgeon’s preference for postoperative breakthrough pain.

#### Index of hematology

Venous blood was collected separately for each patient before surgery (D0) and on the day1 (D1) and day 3 (D3) after surgery to measure levels of the following items: leukocyte count (WBC), neutrophil count (N), lymphocyte count (L), prothrombin time (PT), activated partial thrombin time (APTT), highly sensitive C-reactive protein (hs-CRP), tumor necrosis factor-α (TNF-α), interleukin-1β (IL-1β), IL-6, IL-8, and IL-10.

#### Postoperative pain assessment and follow-up

Pain intensity was evaluated using a visual analog scale (VAS) from 0 cm as no pain to 10 cm as the worst pain imaginable. Patients were assessed for pain intensity separately at rest and coughing at 2, 4, 8, 24, 48, and 72 h post-surgery. The consuming volume and press times of the PCEA pump, the suspicious analgesic-associated adverse reactions, and any postoperative complications within 28 days were recorded.

The patients participating in the clinical trials were requested to complete a questionnaire via telephone 3 months after the surgery. They were questioned about CPSP, and if the answer was positive, the state of CPSP was assessed using the Short-form McGill Pain Questionnaire (SF-MPQ).

### Outcomes

The primary outcome was the proportion of patients with CPSP at 3 months after hepatectomy. The diagnostic criteria of CPSP were referred to the International Association for The Study of Pain (IASP) definition [3]: 1) Pain that develops or increases in intensity after surgical procedure and persists for at least 3 months after surgery. 2) Localized to the surgical field or projected to the innervation territory of a nerve situated around the surgical area. 3) A pain score on the VAS > 1 cm. 4) Pain due to pre-existing pain conditions or infections and malignancy was excluded. Secondary outcomes included the SF-MPQ score in CPSP-positive responders, acute pain intensity within 72 h after surgery, PCEA consumption, postoperative nausea and vomiting score at 24 h (0 = none, 10 = unbearable), perioperative change in hematological indexes, and postoperative complications within 28 days.

### Statistical analysis

The observational data unpublished from patients after liver surgery revealed a CPSP prevalence of 48.6%. Previous studies [16, 17] were used to determine the sample size. Based on an α of 5% and a power of 80%, a sample size of 44 patients per group was sufficient to detect a difference between the parecoxib and placebo groups, given the occurrence rate of 20 and 50%, respectively. In order to allow for 10% early withdrawals and loss to follow-up, 49 patients were sufficient in each group. Finally, we included 105 patients in this study. In addition the two-sided Fisher’s exact test, the power for the primary outcome proportion of patients with CPSP was calculated as > 85%.

The database was established, a two-pass verification was performed using EpiData (version3.1, EpiData Association, Denmark), and data were analyzed using IBM SPSS Statistics (version 22, IBM Corporation, USA). Continuous variables were reported as mean ± standard deviation (SD), and categorical variables were reported as the number (percentage) of patients. The primary outcomes were analyzed based on an intention-to-treat basis according to the previous randomization categories. The proportion of patients developing CPSP between groups was compared using Pearson’s chi-square test. The relative risk (RR) value and its 95% confidence interval (CI) of CPSP were calculated for the parecoxib group. To analyze the sensitivity of the results, the worst-case scenario and per-protocol analysis were operated. For the baseline characteristics and secondary outcomes, normally distributed continuous data were compared using t-test. Non-normally distributed continuous data were compared using the Mann–Whitney U test. The categorical variables were compared using Pearson’s chi-square test or Fisher’s exact test. For repeated measurement, such as inflammation index and VAS within 72 h, an analysis of variance (ANOVA) was employed to assess the between-group difference. To compare the difference in SF-MPQ score between the two groups, we used Mann–Whitney U test. No imputation was performed for missing data for the secondary outcomes.

To investigate other potential risk factors for CPSP after hepatectomy, logistic regression was performed with respect to gender, ASA status, coexisting hypertension, the neutron-lymphocyte ratio (NLR) at baseline, and intervention. The multivariate logistic regression model was constructed after the removal of collinear variables. A type 1 error of 0.05 was used for all analyses.

## Results

**Fig. 1:**
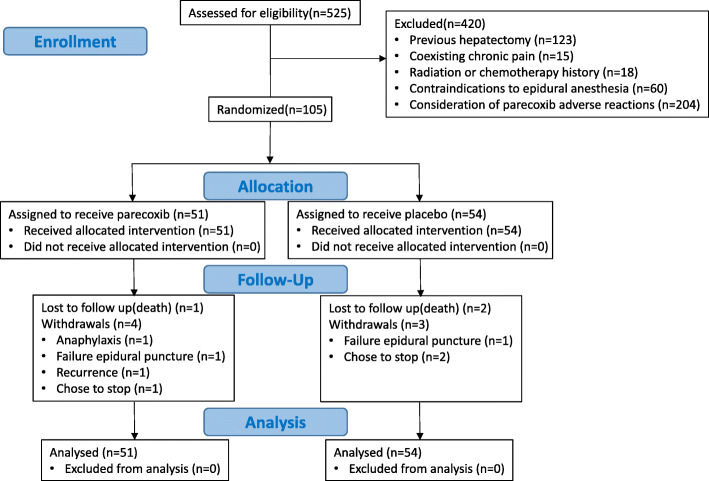
Flow chart

A total of 525 patients were screened, and 105 patients were recruited and assigned to receive intervention from November 2018 to July 2020 (Fig. 1). A total of 95 patients completed the follow-up at 3 months. Among them, 3 patients were lost to follow up due to death within 3 months, 7 patients withdrew from the study before the last assessment: one due to anaphylaxis, two due to failure epidural puncture, one due to recurrence, and three chose to stop. However, data from all the ten patients above were included in the final analysis. The baseline characteristics of the patients were similar between the two groups (Table 1), albeit the parecoxib group had significantly different depression scores from those in the placebo group (*P* = 0.03). Since the cutoff of depression score of the HADS was 8 points, the depression status of the between-group was similar.

**Table 1:** Demographic, baseline, and morphometric characteristics of participants

Factor	Parecoxib Group (***N*** = 51)	Placebo Group (***N*** = 54)	***P*** value
Demographic and baseline			
Age, y	69.9 ± 3.9	70.1 ± 4.2	0.79
Male, No. (%)	41 (80.4)	38 (70.4)	0.23
BMI, kg/m^2^	23.6 ± 2.7	23.0 ± 3.1	0.33
ASA status, No. (%)			0.66
I	22 (43.1)	21 (38.9)	
II	29 (56.9)	33 (61.1)	
History of diabetes, No. (%)	3 (5.9)	10 (18.5)	0.05
History of hypertension, No. (%)	24 (47.1)	26 (48.1)	0.91
HADS- anxiety score, point	2.2 ± 1.7	2.7 ± 2.7	0.73
HADS- depression score, point	1.4 ± 1.4	1.0 ± 1.8	0.03_a_
Surgical characteristics			
TNM stage of hepatocellular carcinoma			0.399
T_1_N_0_M_0_	18 (35.3)	13 (24.1)	
T_2_N_0_M_0_	22 (43.1)	25 (46.3)	
T_3_N_0_M_0_	11 (21.6)	16 (29.6)	
Duration of surgery, h	2.5 ± 0.9	2.4 ± 1.1	0.43
Segment resected, No. (%)			0.07
1	25 (49.0)	25 (46.3)	
2	18 (35.3)	11 (20.4)	
3	8 (15.7)	18 (33.3)	
Pringle manerver duration, min	19.7 ± 12.4	18.9 ± 11.9	0.74
Intraoperative blood loss, mL	245.5 ± 188.8	229.1 ± 182.2	0.65
Intraoperative urine output, mL	277.5 ± 254.4	267.0 ± 233.9	0.83
Fluids volume (crystalloids, colloids), L	2.1 ± 0.6	1.9 ± 0.6	0.18
Intraoperative fentanyl infusion, ug/kg	3.7 ± 0.7	3.6 ± 0.7	0.44

Data are reported as No. (%) or means ± SD as appropriate

*ASA* American Society of Anesthesiologists; *BMI* body mass index; *HADS* Hospital Anxiety and Depression Scale

^a^ Patients in the parecoxib group have significantly different depression scores from those in the placebo group (*P =* 0.03), but the clinical significance was inapparent. Because the cutoff of the depression score in HADS was 8 points, the depression status between-group was similar

### Primary outcomes

**Fig. 2:**
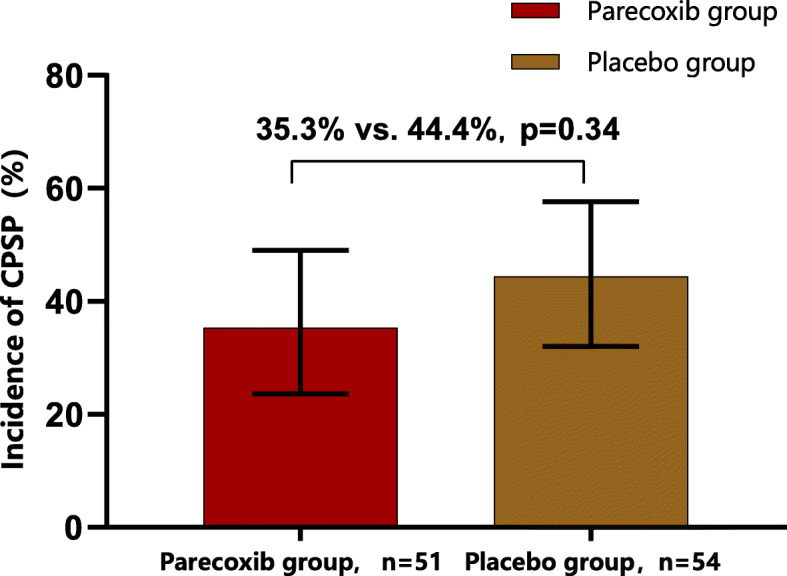
Primary outcomes: incidence of CPSP at 3 months. CPSP, chronic post-surgical pain. Length of black bars for 95% CI of the incidence. For parecoxib group, 95% CI 23.6–49.0%. For the placebo group, 95% CI: 32.0–57.6%

The overall incidence of CPSP at 3 months was 40.0% (42/105) in this cohort. As stated in Table 2 and Fig. 2, perioperative multimodal analgesia with parecoxib did not lower the chance of developing CPSP at 3 months significantly compared to the placebo. The incidence was 35.3% in the parecoxib group and 44.4% in the placebo group, with RR (95% CI) 0.794 (0.493–1.279) for the parecoxib group (*P* = 0.34). The sensitivity analyses, including both worst-case scenario and per-protocol analysis, did not reveal any significant change.

**Table 2:** Primary outcomes: incidence of CPSP at 3 months

Primary Outcomes	Parecoxib group	Placebo group	RR (95% CI)	***P*** value
CPSP at 3 mo, N (%)	18 (35.3)	24 (44.4)	0.794 (0.493 ~ 1.279)	0.34
Worst-case scenario^a^	23 (45.1)	24 (44.4)	1.015 (0.663 ~ 1.552)	0.95
Per-protocol analysis^b^	18 (39.1)	24 (49.0)	0.799 (0.504 ~ 1.265)	0.33

*CPSP* chronic post-surgical pain; *CI* confidence interval; *RR* relative risk

^a^ All patients lost to follow-up in the parecoxib group developed CPSP, while all patients lost to follow-up in the placebo group did not develop the condition

^b^ Only patients who received allocated intervention and completed follow-up were included in his study

### Secondary outcomes

Among the respondents experiencing CPSP at 3 months, no difference was detected in the pain rating index (3.1 ± 2.5 vs. 4.1 ± 3.0, *P* = 0.32) and the present pain intensity (1.4 ± 0.7 vs. 1.6 ± 0.9, *P* = 0.44; Table 3) between parecoxib and placebo groups. However, the VAS for average chronic pain in the parecoxib group was lower than that in the placebo group (1.9 ± 0.7 vs. 2.8 ± 1.4, *P* = 0.04; Table 3). Moreover, 7.4% (4/54) patients developed moderate-to-severe average pain in the placebo group and none in the parecoxib group, albeit not significantly (*P* = 0.122).

**Table 3:** Secondary outcomes in patients receiving parecoxib or placebo analgesia

Secondary Outcomes	Parecoxib Group	Placebo Group	***P*** Value
SF-MPQ			
The Pain Rating Index	3.1 ± 2.5	4.1 ± 3.0	0.32
Sensory subscale	2.1 ± 1.4	2.4 ± 1.8	0.66
Affective subscale	1.0 ± 1.9	1.7 ± 1.6	0.08
Present pain intensity	1.4 ± 0.7	1.6 ± 0.9	0.44
Visual analog scale for average pain, cm	1.9 ± 0.7	2.8 ± 1.4	0.04_*_
Moderate-to-severe pain within 72 h at rest (VAS ≥ 4 cm), N (%)	2 (4.3)	9 (17.3)	0.04_*_
Pain VAS score at rest, cm			< 0.001_*_
2 h	0.1 ± 0.5	0.1 ± 0.3	0.88
4 h	0.2 ± 0.4	0.1 ± 0.3	0.08
8 h	0.2 ± 0.5	0.3 ± 0.9	0.93
24 h	0.6 ± 1.1	1.2 ± 1.3	0.002_*_
48 h	0.3 ± 0.5	1.3 ± 1.4	< 0.001_*_
72 h	0.3 ± 0.6	1.2 ± 1.2	< 0.001_*_
Moderate- to-severe pain within 72 h with coughing (VAS ≥ 4 cm), N (%)	15 (32.6)	38 (73.1)	< 0.001_*_
Pain VAS score with coughing			0.001_*_
2 h	0.4 ± 1.0	0.4 ± 0.9	0.72
4 h	0.7 ± 0.9	0.6 ± 0.7	0.77
8 h	1.2 ± 1.2	1.3 ± 1.6	0.98
24 h	2.0 ± 1.7	3.2 ± 1.8	< 0.001_*_
48 h	1.9 ± 1.3	3.7 ± 2.1	< 0.001_*_
72 h	2.2 ± 1.5	3.6 ± 1.9	< 0.001_*_
Postoperative analgesia			
Total PCEA consumption within 72 h, mL	197.4 ± 43.6	219.2 ± 42.4	0.01_*_
Effective press rate, %	93.3 ± 12.8	93.9 ± 9.7	0.52
Rescue analgesia, time	0.0 ± 0.1	1.2 ± 1.6	< 0.001_*_
Nausea score at 24 h (0 = none to 10 = unbearable)	1.0 ± 2.2	1.3 ± 2.2	0.46
Epidural adverse reactions, N (%)	12(24)	17(31.5)	0.40
Length of stay in hospital after surgery, days	8.3 ± 2.3	8.2 ± 2.5	0.56
Postoperative complications within 28 days, N (%)	6 (11.8)	8 (14.8)	0.65
RR (95% CI)	0.794 (0.296–2.131)	–	
Pleural effusion, N (%)	1 (2)	4 (7.4)	0.36
Ascites, N (%)	1 (2)	2 (3.7)	1.00
Postoperative infection, N (%)	2 (3.9)	1 (1.9)	0.61
Cognitive dysfunction, N (%)	1 (2)	0 (0)	0.49
Urinary retention, N (%)	1 (2)	0 (0)	0.49
Acute pulmonary embolism, N (%)	0 (0)	1 (1.9)	1.00

Data are reported as No. (%) or mean ± SD

*PCEA* patient-controlled epidural analgesia; *RR* relative risk; *SF-MPQ* Short-Form McGill Pain Questionnaire; *VAS* visual analog scale

Asterisks for significance values

**Fig. 3:**
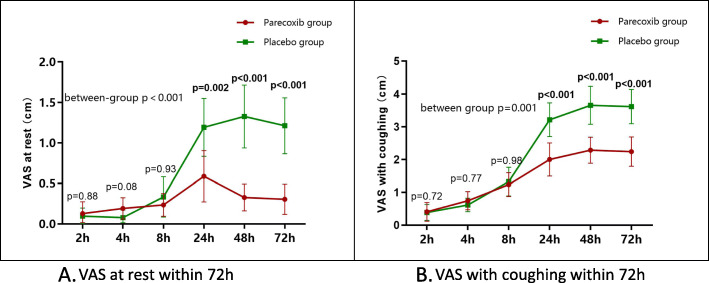
VAS within postoperative 72 h. **A** VAS at rest; **B** VAS with coughing, Spot or square for the mean of the index. Length of bars for standard deviation. *P*-values of inter-group comparisons at each time point indicated as bars. *P*-values for the between-group difference were calculated with repeated measure ANOVA

Table 3 and Fig. 3 show that the postoperative pain intensity in the parecoxib group was significantly higher than that in the placebo group, especially at 24 h, 48 h, and 72 h at rest and with coughing. Moreover, the patients in the placebo group consumed more PCEA volume (219.2 ± 42.4 mL vs.197.4 ± 43.6 mL, *P* = 0.01) and needed more rescue analgesia (0.0 ± 0.1 vs. 1.2 ± 1.6, *P* < 0.001; Table 3) than the PCEA group. However, no differences were observed between the two groups in postoperative analgesia-associated adverse reactions, length of hospital stay after surgery, and postoperative complications within 28 days (Table 3).

**Fig. 4:**
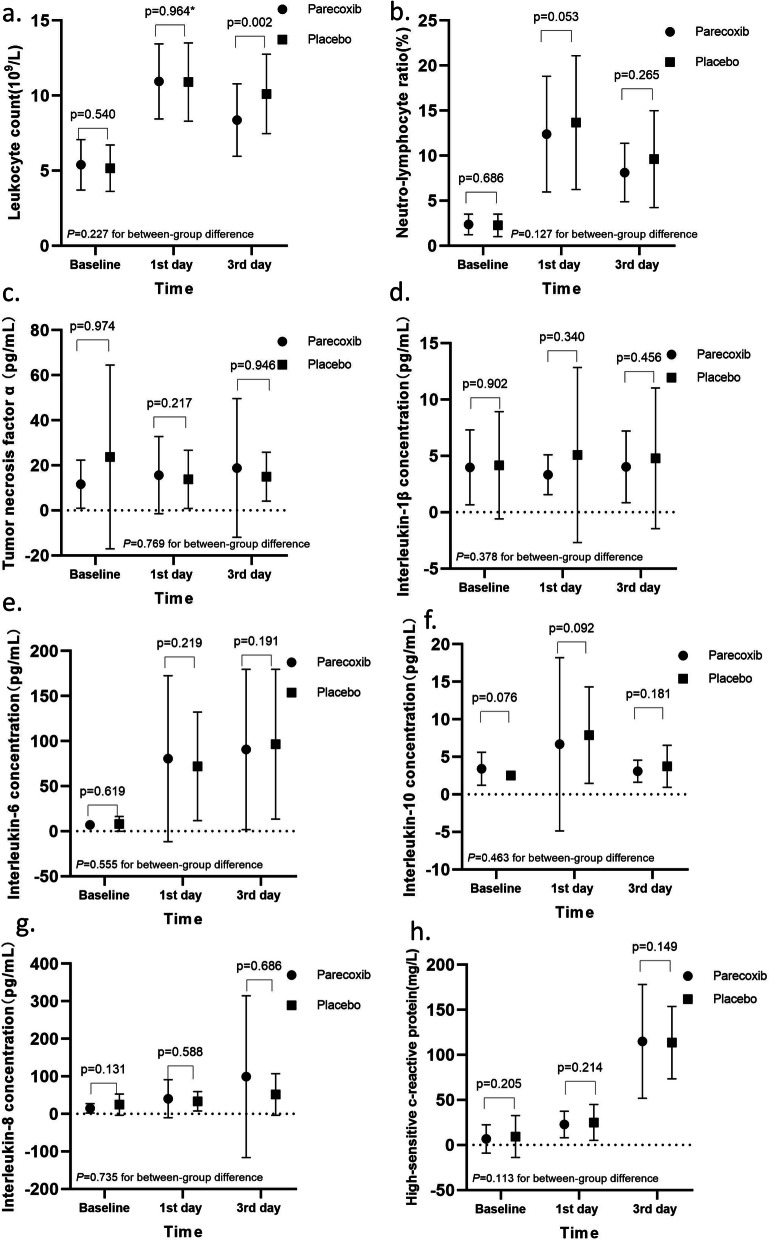
Perioperative change of inflammatory indexes. **a** Leukocyte count change over time; (**b**) Neutron-lymphocyte ratio change over time; (**c**) Tumor necrosis factor-α change over time; (**d**) IL-1β change over time; (**e**) IL-6 change over time; (**f**) IL-10 change over time; (**g**) IL-8 change; (**h**) Highly sensitive CRP over time. Spot or square for the mean of the index. Length of bars for standard deviation. *P*-values of inter-group comparisons at each time point were provided above bars. *P*-values with asterisk were calculated with an independent t-test. Unsigned *P*-values with Mann–Whitney U test. *P*-values for the between-group difference were calculated with repeated measures ANOVA

The perioperative changes in inflammatory indexes are illustrated in Fig. 4 and Table 4. Herein, we did not observe a significant influence of parecoxib than placebo on peripheral inflammatory parameters, including leukocyte count, NLR, hs-CRP, TNF-α, IL-1β, IL-6, IL-8, and IL-10; also, the between-group differences were not significant with respect to the prothrombin time (*P* = 0.262) and activated partial thrombin time (*P* = 0.250).

**Table 4:** Perioperative changes of laboratory data

Indicators	Parecoxib Group	Placebo Group	***P*** Value
Leukocyte count (10^9^/L)			0.227
	*n* = 51	*n* = 54	
Baseline	5.5 ± 1.5	5.4 ± 1.7	0.540
POD 1	11.0 ± 2.8	11.0 ± 2.8	0.964
POD 3	8.7 ± 3.5	10.1 ± 3.0	0.002*
NLR			0.127
	*n* = 51	*n* = 54	
Baseline	2.3 ± 1.1	2.7 ± 2.5	0.686
POD 1	11.7 ± 6.4	13.6 ± 7.0	0.053
POD 3	8.0 ± 4.3	9.0 ± 4.6	0.265
hs-CRP (mg/l)			0.113
	*n* = 45	*n* = 48	
Baseline	5.0 ± 12.4	6.3 ± 16.8	0.205
POD 1	23.4 ± 15.9	31.5 ± 31.4	0.214
POD 3	114.4 ± 54.7	133.8 ± 53.4	0.149
TNF-α (pg/ml)			0.769
	*n* = 39	*n* = 39	
Baseline	14.5 ± 20.1	19.0 ± 33.6	0.974
POD 1	17.8 ± 19.7	11.4 ± 10.4	0.217
POD 3	17.6 ± 26.8	13.9 ± 9.6	0.946
IL-1β (pg/ml)			0.411
	*n* = 46	*n* = 46	
Baseline	3.5 ± 2.8	3.6 ± 3.6	0.902
POD 1	3.0 ± 1.4	4.6 ± 6.9	0.340
POD 3	3.8 ± 3.2	4.4 ± 4.8	0.456
IL-6 (pg/ml)			0.555
	*n* = 46	*n* = 46	
Baseline	5.9 ± 4.8	8.5 ± 13.0	0.619
POD 1	88.3 ± 113.0	73.2 ± 53.4	0.222
POD 3	74.5 ± 72.4	80.6 ± 66.2	0.191
IL-8 (pg/ml)			0.351
	*n* = 38	*n* = 41	
Baseline	21.1 ± 36.5	24.2 ± 27.6	0.131
POD 1	43.9 ± 51.2	34.2 ± 31.3	0.588
POD 3	86.7 ± 197.4	50.5 ± 51.0	0.686
IL-10 (pg/ml)			0.588
	*n* = 38	*n* = 43	
Baseline	3.4 ± 2.2	2.6 ± 0.7	0.076
POD 1	6.5 ± 10.3	7.5 ± 6.0	0.092
POD 3	3.0 ± 1.4	3.9 ± 2.8	0.181
PT (s)			0.262
	*n* = 51	*n* = 54	
Baseline	11.4 ± 0.9	11.3 ± 0.8	0.562
POD 1	13.3 ± 1.3	13.1 ± 1.1	0.367
POD 3	13.8 ± 1.4	13.4 ± 1.3	0.194
APTT (s)			0.250
	*n* = 51	*n* = 54	
Baseline	26.7 ± 1.7	26.4 ± 2.2	0.145
POD 1	27.6 ± 2.9	26.7 ± 2.4	0.174
POD 3	31.6 ± 3.8	31.2 ± 3.0	0.721

Data are reported as mean ± SD

*NLR* neutrophil-lymphocyte ratio; *hs-CRP* highly-sensitive C-reactive protein; *TNF-α* tumor necrosis factor-α; *IL-1β* interleukin-1β; *IL-6*interleukin-6; *IL-8* interleukin-8; *IL-10* interleukin-10; *PT* prothrombin time; *APTT* activated partial thrombin time. Asterisks for significance values

*Post-hoc* analysis using logistic regression for gender, ASA status, coexisting hypertension, the NLR at baseline, and group intervention was summarized in Table 5. In this model, ASA status and coexisting hypertension did not significantly affect the occurrence of CPSP at 3 months. However, male gender and high NLR at baseline were significantly related to developing CPSP in elderly patients after primary hepatectomy.

**Table 5:** Logistic Regression Predicting Likelihood of CPSP at 3 Months

	B	SE	Wald	df	***P*** value	Odds Ratio	95% CI
Parecoxib	0.429	0.450	0.907	1	0.341	1.535	0.635–3.711
Gender	−1.163	0.576	4.082	1	0.043_*_	0.313	0.101–0.966
ASA status	0.722	0.744	0.942	1	0.332	2.058	0.479–8.844
Hypertension	0.560	0.711	0.619	1	0.431	1.750	0.434–7.052
NLR at baseline	0.384	0.180	4.556	1	0.033_*_	1.469	1.032–2.090
Constant	−3.209	1.234	6.758	1	0.009_*_	0.040	

Reference for parecoxib was therapy with parecoxib; reference for gender was male; reference for ASA status was grade I

*B* slope; *CPSP* chronic post-surgical pain; *CI* confidence interval; *df* degrees of freedom; SE, standard error; NLR, neuro-lymphocyte ratio

Asterisks for significance values

## Discussion

In the current study, the overall prevalence of CPSP at 3 months after hepatectomy was 40% (42/105), and moderate-to-severe pain accounted for 3.8% (4/105). This finding was consistent with a single-center observational study reporting a CPSP prevalence of 50% in patients 3 months after liver transplantation [4]. Moreover, with an incision similar to hepatectomy, open cholecystectomy reported an incidence of CPSP varying from 3 to 50% [18]. The difference in CPSP incidence originated from differences in study design or selected study populations [19]. Typically, CPSP is clinically significant after open liver resection in elderly patients, although the pain intensity is mild.

According to the current results, parecoxib could not significantly reduce the prevalence of CPSP, despite an absolute decrease of 9.1%. This difference was less than the 30% difference anticipated while estimating the sample size. These findings were consistent with the previous results in both the worst-case scenario and per-protocol analysis. Helmond et al. reached a similar conclusion for patients after breast cancer surgery [20]. Conversely, the study by Ling et al. showed that parecoxib restrains chronic pain development significantly [21]. However, while interviewing those with chronic pain by SF-MPQ, milder pain intensity was noted in the parecoxib group. Moreover, all the four cases with moderate-to-severe average pain (VAS 4–7 cm), occurred in the placebo group. Therefore, the present study suggested that parecoxib does not reduce the prevalence of chronic post-hepatectomy pain significantly in elderly patients at 3 months but has a potential benefit of reducing the intensity of the chronic pain. Thus, the perioperative use of parecoxib might improve the quality of life in elderly patients with CPSP.

In order to guide the use of parecoxib in clinical practice, the knowledge of pharmacokinetics is essential. Parecoxib is rapidly hydrolyzed by enzymes in the liver after a single intravenous injection and converted into the active metabolite valdecoxib [12]; perceivable analgesia occurs within 10 min, and the maximum effect appears within 2 h. Based on the 8-h half-life of valdecoxib, the plasma concentration can be balanced within 3 days if parecoxib is administered once (40 mg) every 12 h. In the current protocol, parecoxib was administered 10 min before the incision. As a response to stimulus, the analgesic effect is observed, lasting for five half-lives. In order to understand the underlying perioperative effect of parecoxib in the early postoperative period, we recorded a series of changes during the medication period. As confirmed by many studies [9], an uncontrolled acute postoperative pain is a strong predictor of CPSP, provoking central sensitization [8]. In this study, the intensity of acute postoperative pain in the two groups varied following similar trends: the pain intensity was trivial during the first 8 h postoperatively, which gradually increased and reached the peak on day 3 postoperatively. Some studies [17, 22] described the consistent trajectories. We concur that the analgesic effect on the day of surgery was mainly due to sufficient epidural anesthesia. However, 24 h after the surgery, the effect of epidural analgesia was insufficient. Also, the blood concentration of parecoxib reached a steady state, effectively reducing acute postoperative pain. Conversely, a higher percentage of patients in the placebo group experienced moderate-to-severe pain and needed additional PCEA and rescue analgesia. Therefore, the perioperative use of parecoxib based on general anesthesia combined with continuous epidural analgesia has significant advantages in controlling acute pain after hepatectomy in elderly patients.

Based on the analysis of a series of peripheral inflammatory indexes, we deduced the following facts: 1) concentrations of hs-CRP and IL-6 increase gradually over time, the trend coincided with the postoperative pain intensity; 2) peripheral leukocyte count, NLR, and IL-10 increase and reach the peak on day 1 postoperatively, followed by a decrease on day 3; 3) No connection was established between parecoxib and inflammatory changes in peripheral blood. Peng et al. found in aged rats that parecoxib inhibits hepatectomy-induced IL-1β and TNF-α expression in the hippocampus through the downregulation of the COX-2/PGE2 pathway [23]. Bjurstrom et al. [16] reported that the proinflammatory mediators in cerebrospinal fluid are associated with persistent post-surgical pain. In clinical trials, due to technical limitations, real-time monitoring of central neuroinflammation is challenging. Although peripheral inflammatory markers are insensitive to neuroinflammation, our results suggested that 1) level of systemic inflammation may indicate the intensity of acute pain; 2) postoperative inflammatory and anti-inflammatory reactions are conducted simultaneously; 3) the anti-inflammatory effect provided by parecoxib is insufficient to fight with the enormous postoperative inflammatory response that promotes central sensitization. Coincidentally, Turan et al. [24] reported that even with glucocorticoids, the most potent anti-inflammatory drug, CPSP could not be prevented effectively. Therefore, other mechanisms might be involved in the development of central sensitization besides inflammation.

Surprisingly, we found that females had a reduced risk of developing CPSP in elderly patients after hepatectomy. The association between sex and pain has been studied widely. Sorge et al. [25] revealed remarkably different pathways in male and female mice to determine pain hypersensitivity. Hormone levels may play a role in gender differences in pain. We also found that a high preoperative NLR was associated with the development of CPSP. Bugada et al. [26] reported that NLR > 4 is correlated with persistent post-surgical pain after inguinal hernia repair. Other studies [13] revealed that psychological factors, history of pre-existing chronic pain, and preoperative chemotherapy are also predictors of CPSP. Therefore, the condition may have been predetermined before surgery.

The application of NSAIDs in elderly patients has been controversial due to the concerns of severe adverse reactions. NSAIDs-related adverse reactions include myocardial infarction, acute kidney failure, severe gastrointestinal ulceration, anaphylaxis, and coagulopathy. Currently, no significant difference has been detected between the two groups in postoperative complications and coagulation change in our study. Moreover, none of the above side effects were reported. One patient in parecoxib withdrew from the trial because of severe anaphylaxis on day 1 post-surgery. However, given our small sample size, the power to evaluate those side effects is limited. Thus, the safety of parecoxib use in elderly patients requires further verification.

Nevertheless, the present study has several limitations. It was a single-center RCT based on a small sample size. The lack of statistical significance could be attributed to low statistical power due to the small sample size. Due to the potential adverse reactions associated with COX-2 inhibitors, we used stringent inclusion criteria. It inevitably reduced the sample size and affected the generalization of our results. The sample size in this study was smaller than that in Kehlet et al. [24] but similar to that of Anwar et al. However, few patients were lost to follow-up after receiving the assigned intervention, and sensitivity analysis showed that the final results were not affected by the lost cases. Similarly, we evaluated CPSP with SF-MPQ instead of using an objective clinical diagnosis. Investigation on the nature of chronic pain might be limited. In the future, multi-centered RCT for objective assessment with a larger sample size should be conducted to seek a perioperative analgesia strategy and prevent chronic post-surgical pain in elderly patients.

## Conclusions

In conclusion, parecoxib reduced the prevalence of CPSP in elderly patients after hepatectomy under epidural analgesia from 44.4 to 35.3%, with no statistical significance. In addition, parecoxib markedly reduced CPSP intensity and was used to optimize postoperative acute pain management. Thus, prudent but individualized use of parecoxib in healthy elderly patients undergoing liver resection is recommended.

## Supplementary Information

**Additional file 1.**

## Data Availability

The datasets generated during the current study are available in the RESMAN repository, [http://www.medresman.org.cn/pub/cn/proj/projectshshow.aspx?proj=2045].

## Abbreviations

CPSP: Chronic post-surgical pain
PCEA: Patient-controlled epidural analgesia
NSAIDs: Nonsteroidal anti-inflammatory drugs
COX-2: Cyclooxygenase-2
ASA: American society of anesthesiologists
HADS: Hospital anxiety and depression scale
WBC: Leukocyte count
N: Neutrophil count
L: Lymphocyte count
NLR: Neutron-lymphocyte ratio
PT: Prothrombin time
APTT: Activated partial thrombin time
hs-CRP: Highly sensitive C-reactive protein
TNF-α: Tumor necrosis factor-α
IL-1β: Interleukin-1β
IL-6: Interleukin-6
IL-8: Interleukin-8
IL-10: Interleukin-10
VAS: Visual analog scale
SF-MPQ: Short-form McGill pain questionnaire
IASP: International association for the study of pain
IQR: Interquartile range
